# Antimicrobial Susceptibility and Characterization of Extended-Spectrum β-Lactamase-Producing *Escherichia coli* Isolated from Stools of Primary Healthcare Patients in Ethiopia

**DOI:** 10.3390/antibiotics13010093

**Published:** 2024-01-18

**Authors:** Deneke Wolde, Tadesse Eguale, Haile Alemayehu, Girmay Medhin, Aklilu Feleke Haile, Mateja Pirs, Katja Strašek Smrdel, Jana Avberšek, Darja Kušar, Tjaša Cerar Kišek, Tea Janko, Andrej Steyer, Marjanca Starčič Erjavec

**Affiliations:** 1 Department of Medical Laboratory Science, College of Medicine and Health Sciences, Wachemo University, Hossana P.O. Box 667, Ethiopia; denekewolde@gmail.com; 2 Aklilu Lemma Institute of Pathobiology, Addis Ababa University, Addis Ababa P.O. Box 1176, Ethiopia; tadesse.eguale@aau.edu.et (T.E.); haile.alemayehu@aau.edu.et (H.A.); girmay.medhin@aau.edu.et (G.M.); aklilu.feleke@aau.edu.et (A.F.H.); 3 Department of Microbiology, Biotechnical Faculty, University of Ljubljana, 1000 Ljubljana, Slovenia; 4 Institute of Microbiology and Immunology, Faculty of Medicine, University of Ljubljana, 1000 Ljubljana, Slovenia; mateja.pirs@mf.uni-lj.si (M.P.); katja.strasek@mf.uni-lj.si (K.S.S.); 5 Institute of Microbiology and Parasitology, Veterinary Faculty, University of Ljubljana, 1000 Ljubljana, Slovenia; jana.avbersek@vf.uni-lj.si (J.A.); darja.kusar@vf.uni-lj.si (D.K.); 6 National Laboratory of Health, Environment and Food, 2000 Maribor, Slovenia; tjasa.cerar.kisek@nlzoh.si (T.C.K.); tea.janko@nlzoh.si (T.J.); andrej.steyer@nlzoh.si (A.S.)

**Keywords:** *Escherichia coli*, antimicrobial susceptibility, ESBL, Ethiopia, whole-genome sequencing

## Abstract

Antimicrobial resistance of *Escherichia coli* is a growing problem in both developed and developing countries. This study aimed to investigate the phenotypic antimicrobial resistance of *E. coli* isolates (*n* = 260) isolated from the stool specimen of patients attending public health facilities in Addis Ababa and Hossana. This study also aimed to characterize phenotypically confirmed extended-spectrum beta-lactamase (ESBL)-producing *E. coli* isolates (*n* = 22) using whole-genome sequencing. Resistance to 18 different antimicrobials was assessed using the disc diffusion method according to the European Committee on Antimicrobial Susceptibility Testing (EUCAST) guidelines. The highest resistance rate among the *E. coli* isolates was found for ampicillin (52.7%), followed by trimethoprim-sulfamethoxazole (29.6%). Of all isolates, 50 (19.2%) were multidrug-resistant and 22 (8.5%) were ESBL producers. ESBL genes were detected in 94.7% of the sequenced *E. coli* isolates, and multiple β-lactamase genes were detected in 57.9% of the isolates. The predominant ESBL gene identified was *bla*_CTX-M-15_ (78.9%). The *bla*_TEM-1B_ gene was detected in combination with other ESBL genes in 57.9% of the isolates, while only one of the sequenced isolates contained the *bla*_TEM-1B_ gene alone. The *bla*_CTX-M-3_ gene was detected in three isolates. The genes *bla*_CTX-M-15_ and *bla*_TEM-1B_ as well as *bla*_CTX-M-15_ and *bla*_TEM-169_ were confirmed to coexist in 52.6% and 10.5% of the sequenced *E. coli* isolates, respectively. In addition, *bla*_OXA-1_ was identified together with *bla*_CTX-M-15_ and *bla*_TEM-1B_ in one isolate, and in one isolate, *bla*_TEM-169_ together with *bla*_CTX-M-15_ and *bla*_TEM-1B_ was found. The results obtained show that measures need to be taken to reduce the spread of drug resistance and ensure the long-term use of available antimicrobials.

## 1. Introduction

Antimicrobial resistance (AMR) is a serious public health problem. It is rapidly becoming one of the major health problems that could seriously affect the functioning of health systems [1]. Every year, increasing numbers of multidrug-resistant (MDR) bacterial strains cause life-threatening infections and the deaths of thousands of people [2]. The World Health Organization’s (WHO) global report on AMR has shown that bacterial resistance to common antimicrobials has reached alarming levels in many parts of the world, suggesting that many of the available treatments for common infections are becoming ineffective in some areas [3]. In 2019, an estimated 4.95 million deaths associated with bacterial AMR strains were reported [4]. Six pathogens were responsible for 73.4% of deaths attributable to bacterial AMR strains. The most prevalent pathogen among the six was *Escherichia coli* [4]. *E. coli* is one of the most important human pathogens known to cause a wide range of intestinal and extra-intestinal infections. AMR in *E. coli* is reported worldwide, and the increasing resistance rates in *E. coli* is a growing problem in both developed and developing countries [5]. *E. coli* and other members of the *Enterobacteriaceae* family are on the WHO list of antibiotic-resistant “priority pathogens” that pose the greatest threat to human health [6]. The antimicrobial resistance of *E. coli* in developing countries, including Ethiopia, is a major burden for patients and healthcare systems. Studies conducted in Ethiopia showed high rates of MDR *E. coli* [7]. More than 50% of *E. coli* isolates are resistant to commonly used antimicrobials (including third-generation cephalosporins) [8].

The most common mechanism by which bacteria acquire resistance to β-lactam antibiotics is through the expression of β-lactamases. Currently, over 1150 chromosomal, plasmid- and transposon-mediated β-lactamase genes are known [9]. Critical β-lactamases are enzymes whose genes are encoded on mobile genetic elements that are transferable from strain to strain and between bacterial species. The three major categories of β-lactamases are plasmid-mediated extended-spectrum β-lactamases (ESBLs), AmpC cephalosporinases and carbapenemases [10]. *E. coli* is one of the predominant ESBL-producing *Enterobacteriaceae* strains [11]. ESBL production in *E. coli* is usually associated with resistance genes encoded by *bla*_TEM_, *bla*_SHV_ and *bla*_CTX-M_ [12]. Currently, more than 187 variants of TEM types, more than 141 variants of SHV, and 119 variants of CTX-M have been identified [9]. The number of ESBL-producing *E. coli* has increased enormously worldwide, and infections associated with these strains are a major problem in clinical practice due to the limited therapeutic options for their treatment [13]. The prevalence of ESBL-producing pathogens in Ethiopia is alarmingly high [14]. The prevalence of ESBL-producing bacteria varies between 41.2% and 59.0% [15,16,17]. As carbapenems and other alternative antimicrobials are expensive and difficult to find in developing countries, infections caused by ESBL-producing bacteria often result in a high mortality rate [16]. According to Tufa et al., the mortality rate for infections caused by ESBL-producing bacteria is 86% [16]. Understanding the burden of AMR is critical to making informed decisions, particularly with regard to antimicrobial stewardship, and could help to develop guidelines for the empirical treatment of *E. coli*. In addition, *E. coli* is known to serve as a reservoir for several antimicrobial resistance genes and is capable of horizontally transferring these genes to other pathogenic and commensal organisms. Therefore, understanding the antimicrobial susceptibility of *E. coli* may provide an indication of the burden of antimicrobial resistance in other Gram-negative organisms circulating in a given community [5]. However, there are limited data on the antimicrobial susceptibility patterns and genomic characteristics of ESBL-producing *E. coli* in Ethiopia, although *E. coli* resistance to common antimicrobial agents used for treatment continues to increase. The aim of this study was to evaluate the phenotypic antimicrobial susceptibility of *E. coli* from patients attending public health centers in Addis Ababa city and Hossana town, Ethiopia, and to bioinformatically characterize ESBL-producing isolates based on the whole-genome sequences obtained.

## 2. Results

### 2.1. Study Participants

Table 1 lists the sociodemographic characteristics of the patients from whom *E. coli* isolates were obtained. The median income and age of participants with an interquartile range are 106.38 (70.92–153.37) USD and 22 (9–32) years, respectively.

### 2.2. Antimicrobial Susceptibility of E. coli Isolates

The antimicrobial susceptibility of the studied *E. coli* isolates is summarized in Table 2 and Table S1. One hundred and nineteen (45.8%) *E. coli* isolates were susceptible to all antimicrobials tested: seventy-one (48.3%) from Addis Ababa and forty-eight (42.5%) from Hossana. The highest resistance rates among the *E. coli* isolates were found for ampicillin and trimethoprim-sulfamethoxazole, followed by amoxicillin-clavulanic acid, cefuroxime, ceftriaxone, and cefotaxime. A very high percentage (86.9%) of *E. coli* strains were sensitive to increased exposure to cefuroxime. The *E. coli* isolates in the patients from Hossana town showed a higher resistance rate to all other antimicrobials, except for ampicillin and amoxicillin-clavulanic acid. All isolates were susceptible to carbapenems (ertapenem, imipenem, and meropenem) and amikacin. In addition, all *E. coli* isolates from Addis Ababa were susceptible to tobramycin, in contrast to 2.7% of tobramycin-resistant isolates from Hossana.

Chi-square analysis revealed a statistically significant difference in antimicrobial susceptibility patterns in Addis Ababa and Hossana (*p* < 0.05). Resistance to aztreonam (*p* = 0.045), ciprofloxacin (*p* = 0.023), cefotaxime (*p* = 0.033), cefepime (*p* = 0.041), and levofloxacin (*p* = 0.020) was significantly higher in Hossana than in Addis Ababa (Table 2).

### 2.3. Antimicrobial Resistance Patterns of E. coli Isolates

The antimicrobial resistance patterns observed in the *E. coli* tested are summarized in Table 3. Resistance to one or more antimicrobials was detected in 54.2% of the isolates. Of the resistant isolates, 24.1% were resistant to a single agent, such as ampicillin and trimethoprim-sulfamethoxazole. MDR was detected in 50 (19.2%) isolates. Resistance to six or more antimicrobials was detected in 27 (10.4%) isolates: 16 from Hossana and 11 from Addis Ababa. Thirteen (5.0%) isolates were resistant to all cephalosporins tested. The results of this study showed that the proportion of MDR in *E. coli* was significantly higher in isolates from Hossana than in isolates from Addis Ababa (*X*^2^ = 5.27, *p* = 0.022). Nearly 62% of MDR isolates were from Hossana town. Males and those in the age group between 20 and 45 years contributed to 59.2% and 44.9% of the MDR *E. coli* isolates, respectively. Of the MDR isolates, 100.0% were resistant to ampicillin, 75.5% were resistant to trimethoprim-sulfamethoxazole, 71.4% were resistant to ceftriaxone, and 69.4% were resistant to cefotaxime. The isolates had widely varying resistance patterns. A total of 33 resistance patterns were observed among the *E. coli* isolates: 20 in Addis Ababa, 25 in Hossana, and 12 common to both. The most frequently detected MDR pattern was AM, AMC, SXT, followed by AM, CIP, LVX, SXT (Table 3).

### 2.4. ESBL-Producing E. coli Isolates

This study found that 22 (8.5%) of the strains were ESBL producers. ESBL-producing *E. coli* showed 100% resistance to ampicillin, ceftriaxone, and cefuroxime and 95.5% resistance to cefotaxime. In addition, ESBL-producing strains showed 22.7–63.6% resistance to amoxicillin-clavulanic acid, levofloxacin, ciprofloxacin, aztreonam, ceftazidime, cefepime, and trimethoprim-sulfamethoxazole. ESBL-producing *E. coli* showed the lowest resistance to piperacillin-tazobactam (9.1%), gentamicin (9.1%), and tobramycin (4.5%). Of the ESBL-producing *E. coli* strains, 59.1% were from the Hossana site. However, there was no significant difference in the distribution of ESBL-producing *E. coli* between the strain from Addis Ababa and Hossana (*X*^2^ = 2.39, *p* = 0.122). Sixteen resistance patterns were observed among the ESBL-producing *E. coli* strains for the tested antimicrobials (Table 3).

### 2.5. Whole-Genome Sequencing Analysis of ESBL-Producing E. coli Isolates

Of the 22 phenotypically confirmed ESBL-producing *E. coli* isolates, 19 were subjected to whole-genome sequencing on the Illumina platform to identify the ESBL gene profile (unfortunately, three strains could not be revived). All assemblies passed the QC threshold of N50 > 15 Kb and <500 contigs. At least one known ESBL gene was detected in 94.7% of the sequenced isolates, and 57.9% harbored two or more β-lactamase genes. The predominant ESBL gene identified was *bla*_CTX-M-15_ (78.9%). The *bla*_TEM-1B_ gene was detected in combination with other ESBL genes in 57.9% of the isolates, while only one of the sequenced isolates contained the *bla*_TEM-1B_ gene alone. The *bla*_CTX-M-3_ gene was detected in three isolates. In this study, the coexistence of *bla*_CTX-M-15_ and *bla*_TEM-1B,_ as well as of *bla*_CTX-M-15_ and *bla*_TEM-169,_ was confirmed in 52.6% and 10.5% of the sequenced *E. coli* isolates, respectively. In addition, *bla*_OXA-1_ was identified together with *bla*_CTX-M-15_ and *bla*_TEM-1B_ in one isolate, and *bla*_TEM-169_ was confirmed together with *bla*_CTX-M-15_ and *bla*_TEM-1B_ in one isolate. Eight (80.0%) of the ten ESBL-producing *E. coli* isolates from Hossana harbored one or two β-lactamase genes in addition to *bla*_CTX-M-15_ (Table 4).

## 3. Discussion

Antimicrobial resistance is one of the greatest threats to humanity in the 21st century [18]. This study investigated the antibiotic resistance profiles of *E. coli* isolated from the stools of patients and characterized ESBL-producing *E. coli* isolates. Unfortunately, we were not able to obtain any information about the clinical situation or diagnosis of patients, which is a limitation of the study.

### 3.1. Antimicrobial Resistance of Studied E. coli Isolates

This study found that 54.1% of *E. coli* strains were resistant to at least one or more of the antimicrobial agents tested. This is comparable to previous reports of pathogenic *E. coli* strains isolated from children in Wolaita Sodo, southern Ethiopia [19], and a meta-analysis in Ethiopia [7], where 61.7% and 45.4% of strains were resistant to at least one antimicrobial agent, respectively.

Ampicillin and trimethoprim-sulfamethoxazole are widely used antimicrobial agents for the treatment of infections in humans and animals. The isolates from the present study had high resistance to ampicillin (52.7%), which is comparable to other studies on *E. coli* obtained from outpatients in hospitals in Lagos State, from animal handlers in abattoirs, poultry farmers, and open markets in Lagos, Nigeria (59.1%) [20], and from poultry farmers in Lusaka, Zambia (46.8%) [21]. However, it is lower compared to previous reports of pathogenic *E. coli* strains isolated from children in Wolaita Sodo, southern Ethiopia (70.6%) [19], diarrheagenic *E. coli* isolates in Trans-Nzoia County, Kenya (83.9%) [22], and *E. coli* isolated from diarrheic patients in public health centers in Eastern Cape, South Africa (88%) [23]. In addition, our study showed a high percentage (29.6%) of *E. coli* isolates resistant to trimethoprim-sulfamethoxazole, which is lower than the resistance rate from previous studies on *E. coli* from children under five years of age with diarrhea at Sodo Christian Hospital in Wolaita Sodo, southern Ethiopia (67.6%) [19], in patients at Kitale County Referral Hospital in Trans-Nzoia County, Kenya (95.7%) [22], in diarrheic patients at a public health center in Eastern Cape, South Africa (78%) [23], in outpatients at hospitals in Lagos State and among livestock farmers at abattoirs, poultry farmers, and open markets in Lagos, Nigeria (61.5%) [20], and among poultry farmers in Lusaka, Zambia (48.3%) [21]. This could be due to the fact that the genes responsible for ampicillin and trimethoprim-sulfamethoxazole resistance probably co-occur due to the same mobile genetic elements [24,25]. As a result, resistance to multiple antimicrobials develops simultaneously, leading to a high level of resistance.

Relatively low proportions of *E. coli* isolates in the current study were resistant to amoxicillin-clavulanic acid (14.2%). However, in previous studies, a high percentage of resistance to amoxicillin-clavulanic acid was found in *E. coli* from diarrheic patients in Ethiopia (64.4%) [26] and in *E. coli* from different clinical samples from patients in different teaching hospitals in Sudan (50.4%) [27]. This may be due to the fact that amoxicillin-clavulanic acid is the most commonly used antibiotic for self-medication and is widely prescribed by healthcare providers. It is inexpensive and is considered first-line therapy in many low- and middle-income countries [28,29,30].

In this study, resistance to levofloxacin was detected in 6.9% of isolates and to ciprofloxacin in 7.3% of isolates. This is comparable to the rate of resistance to ciprofloxacin reported in a study on *E. coli* from Congolese students in Madibou, Brazzaville (4%) [31], and children under five years of age in a study in the pediatric clinic of the commune of Abomey-Calavi, Benin (9.5%) [32]. In contrast, a study conducted in Nigeria found a high percentage of resistance to ciprofloxacin in humans (21.9%), among poultry workers, chickens, and their environment in poultry farms/markets [33]. This could be due to the fact that fluoroquinolones are one of the most commonly prescribed antimicrobial classes in human and veterinary medicine in Nigeria, which could be responsible for the selection pressure that favors the development of quinolone resistance in *E. coli* isolates [34].

Aztreonam is among the last-resort antibiotics currently available for the treatment of these infections [35]. In the present study, 7.7% of the *E. coli* isolates were resistant to aztreonam. In contrast, in a study conducted in the pediatric clinic of the commune of Abomey-Calavi in Benin, children under five years of age showed 100% resistance to aztreonam [32]. This indicates a serious threat to human health as very few effective antibiotics are available for the clinical treatment of infections caused by aztreonam-resistant strains.

The resistance of *E. coli* to gentamicin (1.9%) in this study was consistent with previous findings in Eastern Cape, South Africa, from diarrheic patients attending a public health center (2%) [23]. In addition, resistance to tobramycin in this study was 1.2%, which was lower than the resistance rate found in an Egyptian study on *E. coli* strains isolated from children with diarrhea from Assiut Children’s Hospital (68%) [36]. Interestingly, no resistance to carbapenems (ertapenem, meropenem, and imipenem) and amikacin was detected in the current study. In contrast, resistance to imipenem (1%) and amikacin (3%) was detected in *E. coli* from diarrheic patients attending a public health center in Eastern Cape, South Africa [23]. The most important factors contributing to the emergence of resistance to these antibiotics could be the availability and use of these antimicrobials in the treatment of infectious diseases in South Africa.

Cephalosporins are one of the limited available therapies for the treatment of severe bacterial infections in humans. The third, fourth, and fifth-generation cephalosporins are classified by the WHO as “critically important antimicrobials” in human medicine [37]. However, they are among the most frequently prescribed drugs for the treatment of infections caused by *Enterobacteriaceae*, which increases the selection pressure for resistant organisms. Resistance rates to cefepime (1.6%) in *E. coli* isolated from outpatients in Lagos State hospitals (Nigeria) and from animal caretakers in abattoirs, poultry farms, and open markets (1.6%) [20], and resistance rates to cefotaxime in Lusaka, Zambia (8.6%) in *E. coli* isolated from poultry farmers [21], were almost as low as in this study. However, high rates of resistance to this class of antibiotics were found in previous studies conducted in Benin on *E. coli* isolated from children under five years of age in the pediatric clinic in the commune of Abomey-Calavi (cefotaxime, 100%) [32], and *E. coli* isolated from Congolese students in Madibou, Brazzaville (ceftazidime, 65%) [31].

### 3.2. MDR and ESBL-Producing E. coli among Studied Isolates

ESBL-producing *E. coli* are a major cause of antimicrobial-resistant infections and pose a major threat worldwide as they can cause infections that are difficult to treat in animals and humans [38]. The overall percentage of ESBL-producing *E. coli* strains in this study was 8.5%. This result is lower than previous reports of ESBL-producing *E. coli* in children hospitalized in Ghana (61%) [39]. This could be due to differences in antimicrobial use among the regions or the study population. All children who participated in the Ghanaian study had contact with free-roaming chickens in town, and chicken meat was part of their regular diet. ESBL-producing bacteria are commonly isolated from poultry [39]. The genetic linkage of ESBL-producing *E. coli* between poultry and human populations was also demonstrated in the same study, which could indicate a potential risk of transmission of ESBL-producing bacteria between poultry products and humans and increase the incidence rate in the study population [39].

The MDR rate in the current study was 19.2%, of *E. coli,* which is comparable to the result of a previous study in Ethiopia on *E. coli* isolates from diarrheic patients at Selam Health Center, Addis Ababa (15.1%) [26] and *E. coli* from poultry farmers in Lusaka, Zambia (29.3%) [21]. The result of the current study is very low compared to a meta-analysis of resistance patterns of Gram-negative bacterial pathogens in Ethiopia (78.2%) [40] and other studies on *E. coli* isolated from the stool of hospitalized patients in Kenya [22] and community outpatients and animal caregivers in Lagos, Nigeria (69.6%) [20]. The difference might be related to the study population, sample size, and geographical location. Unlike the current study, where patients are those who come from the community, other studies were conducted among hospitalized patients with a high chance of being exposed to MDR organisms.

A higher percentage of MDR (61.2%) and ESBL-producing *E. coli* (59.1%) in the current study was obtained among the Hossana isolates, in which agricultural and animal husbandry are the common practices of the community. This could be related to the imprudent use of antimicrobials in humans and agriculture, which is considered to be one of the most important factors contributing to the emergence and spread of resistant bacteria [41]. The low socioeconomic status in Hossana, poor sanitation, and unhygienic conditions may also have contributed to the easy circulation of resistant organisms and resistant genetic markers. The inappropriate use of antimicrobials in agriculture can lead to selection pressure on microorganisms in food, water, and the environment, which can serve as a source of infection with MDR organisms for humans and animals [42].

The *bla*_CTX-M-15_ gene is the most widely distributed gene encoding ESBLs associated with human infections [43]. It is mainly found in international high-risk clones [44]. This gene was the dominant genotype among the ESBL-positive isolates in our study. This is in line with the report from Ethiopia, where a study investigated the genome-based epidemiology of ESBL-producing *E. coli* among patients seeking medical care at a tertiary hospital in Jimma [45] and a study on the fecal carriage of ESBL-producing *Enterobacteriaceae* in young children in Tanzania [46]. In addition, the predominance of *bla*_CTX-M-15_ in *E. coli* was also found in bacteriemic patients in two teaching hospitals in Bamako, Mali [47]. These higher rates of the *bla*_CTX-M-15_ gene may be associated with incompatibility group FII conjugative plasmids which carry this gene and play a great role in the spread and acquisition of resistance genes in *E. coli* [48]. In contrast to the present study, *bla*_TEM_ was found to be the predominant β-lactamase gene in a study with children under five years of age in Ethiopia [49] and in diarrheic patients in Ghana [50]. Several variants of the *bla*_CTX-M_ and *bla*_TEM_ genes have been identified on both plasmids and chromosomes [9,51]. ESBL genes located on plasmids spread rapidly between and within bacterial species, whereas ESBL genes integrated into chromosomes are mostly stable and persist without antibiotic selection pressure [51].

*E. coli*-encoding *bla*_CTX-M-15_ is often characterized as multidrug-resistant and a co-producer of OXA-1 or TEM-1B [52,53]. The co-existence of *bla*_CTX-M-15_ and *bla*_TEM-1B_ was observed in 52.6% of *E. coli* with ESBL genes in this study. In agreement with the current study, the coexistence of these genes in *E. coli* isolates was found in a study in Burkina Faso in ESBL-producing *Enterobacteriaceae* among clinical isolates [54] and in Cameroon in ESBLs in *Enterobacteriaceae* isolates in the community [55]. The *E. coli* isolates in our study, which possess *bla*_CTX-M-15_ genes, also encode *bla*_OXA-1_ and *bla*_TEM-169_ β-lactamase genes. This finding is consistent with a previous study from Pakistan that investigated the frequency of resistance to third-generation cephalosporin and the distribution of key genetic determinants of ESBL-producing clinical isolates. This study showed the co-existence of *bla*_CTX-M-15_, *bla*_OXA-1,_ and *bla*_TEM-1_ in 7% of ESBL-producing *E. coli* [56]. The result showed a high rate of co-existence of β-lactamase genes in isolates from Hossana compared to isolates from Addis Ababa, suggesting a high rate of co-selection of plasmids with different resistance genes due to the overuse of antimicrobials in Hossana.

## 4. Conclusions

Resistance to ampicillin and trimethoprim-sulfamethoxazole was high in the current study, and none of the isolates were resistant to carbapenems and amikacin. A high rate of ESBL-producing *E. coli* strains was detected, most of which were encoded by *bla*_CTX-M-15_ and a combination of other ESBL genes in *E. coli*. Overall, MDR- and ESBL-producing isolates were frequently detected among the isolates from Hossana, indicating a high level of irrational use of antimicrobials, leading to high selection pressure. These findings suggest that prudent use of antimicrobials is advisable to reduce the burden of drug resistance and ensure the long-term use of available antimicrobials.

## 5. Materials and Methods

### 5.1. Study Design and Sample Collection

A health institution-based cross-sectional study was conducted in Addis Ababa city and Hossana town. Addis Ababa is the capital of Ethiopia, and it has an estimated population of 5,461,000. The city is home to 23.8% of all urban residents in Ethiopia, and it has an estimated density of 5936 per square kilometer. The majority of the population, especially the urban poor, lives in a slum area of Addis Ababa, and they are exposed to water and sanitation-related diseases [57]. The other study area, Hossana town, is located in the Hadiya zone, 230 km southwest of Addis Ababa, with a total estimated population of 75,963. Hadiya Zone is one of the central zones in the central Ethiopia region. The zone is one of the most densely populated areas in Ethiopia. The population density of the zone is 415 people per square kilometer.

Seventeen public health centers (primary healthcare units) in Ethiopia (thirteen randomly selected health centers from Addis Ababa city and all four health centers from Hossana town) were included in this study. This study included patients of all ages who visited the health centers at the time of data collection and had not taken any antimicrobial agents in the fifteen days prior to the study. Patients who met these inclusion criteria were informed of the purpose and procedures of the study and gave their informed consent before providing a fresh stool sample in a dry, sterile container with Cary–Blair transport medium. A total of 260 samples were collected from December 2021 to September 2022. Each stool sample was carefully labeled with a unique identifier and placed in a cool box (4 °C) with cold packs for transport to the microbiology laboratory for isolation of *E. coli*. Unfortunately, we were not provided with any information about the clinical situation and the diagnosis of patients.

### 5.2. Isolation and Identification of E. coli

For the isolation of *E. coli*, approximately 1 g of stool sample was pre-enriched in 9 mL of buffered peptone water (BPW) (Becton-Dickinson, Sparks, MD, USA) and incubated overnight at 37 °C. It was then streaked on eosin methylene blue agar (EMB) (Oxoid, Basingstoke, UK), and the plates were incubated at 37 °C for 24 h. Colonies that exhibited a metallic green sheen were further examined with biochemical tests (indole, citrate, and urease). A reference strain, *E. coli* ATCC 25922, was used as a positive control for biochemical analysis. After confirmation, *E. coli* isolates (only one from each stool sample) were frozen in a nutrient broth containing 20% glycerol and stored. A total of 260 strains were randomly selected using a computer-generated random sampling technique and exported on slant agars to Slovenia for further analysis, where each strain was streaked on MacConkey agar (BD MacConkey II Agar, Becton Dickinson, Germany) for revitalization. Matrix-assisted laser desorption ionization time of flight (MALDI-TOF) mass spectrometry (Microflex LT with regularly updated BruckerMS library, Brucker Daltonics, Bremen, Germany) was used to confirm *E. coli* before antimicrobial susceptibility testing.

### 5.3. Antimicrobial Susceptibility Testing

Antimicrobial susceptibility testing was performed using the disk diffusion method according to the European Committee on Antimicrobial Susceptibility Testing (EUCAST) guidelines [58]. The following antimicrobial discs were used: ampicillin (Am 10 μg), amoxicillin-clavulanic acid (AMC 20/10 μg), piperacillin-tazobactam (TZP 30/6 μg), cefuroxime (CXMP 30 μg), cefotaxime (CTX 5 μg), ceftriaxone (CRO 30 μg), ceftazidime (CAZ 10 μg), cefepime (FEP 30 μg), ertapenem (ETP 10 μg), meropenem (MEM 10 μg), imipenem (IPM 10 μg), gentamicin (GM 10 μg), amikacin (AN 30 μg), ciprofloxacin (CIP 5 μg), levofloxacin (LVX 5 μg), trimethoprim-sulfamethoxazole (SXT 1.25/23.75 μg), aztreonam (ATM 30 μg), and tobramycin (NN 10 μg). The results were interpreted according to EUCAST guidelines [59]. Multidrug resistance (MDR) was defined as resistance to at least one agent in three or more antimicrobial groups [60]. Extended-spectrum β-lactamase (ESBL) production among the strains was phenotypically determined using the double disk diffusion method suggested by the EUCAST [61]. The presence of ESBL was confirmed by a synergy test between a disk with amoxicillin-clavulanic acid and cephalosporins (cefotaxime and ceftazidime).

### 5.4. DNA Extraction and Whole-Genome Sequencing

Whole-genome sequencing was performed for 21 phenotypically confirmed ESBL-producing *E. coli* isolates. Genomic DNA was extracted using the QIAamp DNA Mini Kit (Qiagen, Hilden, Germany) [62] or STARMag 96 × 4 Universal Cartridge Kit (Seegene Inc. Walnut Creek, CA, USA) [63]. Genomic libraries were prepared using the Illumina DNA Prep (Illumina, San Diego, CA, USA). Isolates were sequenced on the NextSeq 2000 system (Illumina) using 2 × 149 bp paired-end reads chemistry [64].

#### 5.4.1. Raw Data Pre-Processing

Trimmomatic was used to trim raw reads from adapter sequences and low-quality reads [65]. The quality of both raw and trimmed reads was assessed using FastQC v0.11.9 [66]. Species identification was confirmed using KmerFinder v3.0.2 based on trimmed reads.

#### 5.4.2. De Novo Assembly and QC

The assembly of trimmed reads into contigs was carried out with SPAdes v3.15.3 [67] using the default K-Kmer values and the “--isolate” parameters. Quast v5.2.0 was used for the quality assessment of the assemblies [68]. To determine the coverage of the assembly, fastq files were converted into .bam using samtools. Subsequently, «samtools depth» was used to obtain base coverages, which were then averaged and reported.

#### 5.4.3. AMR Identification

Resistance genes were detected with ResFinder 4.1 [69] with the default parameters (80% identity over 60% of the length of the target gene), using assembled sequences as input. Gene prediction was confirmed if the assembled sequence had at least 97% nucleotide match and 100% coverage with genes in the curated *Escherichia coli* database.

### 5.5. Statistical Analysis

Descriptive statistical analysis was performed using WHONET software version 2022, and a chi-square test was used to investigate the association between different variables using SPSS version 25.0. A *p*-value of <0.05 was considered an indicator of statistically significant association.

## Figures and Tables

**Table 1:** Sociodemographic characteristics of the study participants.

Characteristics	Response Category	Number (%)
Location of the health facilities		
	Addis Ababa	147 (56.5)
	Hossana	113 (43.5)
Sex		
	Female	110 (42.3)
	Male	150 (57.7)
Age group		
	0–4 years	27 (10.4)
	5–9 years	43 (16.5)
	10–14 years	31 (11.9)
	15–19 years	21 (8.1)
	20–45 years	121 (46.5)
	46–65 years	16 (6.2)
	>65 year	1 (0.4)
Marital status		
	Single	164 (63.1)
	Married	95 (36.5)
	Divorced	1 (0.4)

**Table 2:** Antimicrobial susceptibility of studied *E. coli* isolates.

Antibiotic	Antibiotic Class	S (Susceptible, Standard Dosing Regimen)			I (Susceptible, Increased Exposure)			R (Resistant)		
		Total Isolates (*n* = 260)	Addis Ababa Strains (*n* = 147)	Hossana Strains (*n* = 113)	Total Strains (*n* = 260)	Addis Ababa Strains (*n* = 147)	Hossana Strains (*n* = 113)	Total Strains (*n* = 260)	Addis Ababa Strains (*n* = 147)	Hossana Strains (*n* =113)
		*n* (%)	*n* (%)	*n* (%)	*n* (%)	*n* (%)	*n* (%)	*n* (%)	*n* (%)	*n* (%)
AM	Penicillin	123 (47.3)	73 (49.7)	50 (44.2)	0	0	0	137 (52.7)	74 (50.3)	63 (54.3)
AMC	β-lactam + inhibitors	223 (85.8)	123 (83.7)	100 (88.5)	0	0	0	37 (14.2)	24 (16.3)	13 (11.5)
TZP	Antipseudomonal penicillin + β-lactam inhibitors	257 (98.8)	145 (98.6)	112 (99.1)	0	0	0	3 (1.2)	2 (1.4)	1 (0.9)
CXMp	Non-extended spectrum cephalosporins	0	0	0	226 (86.9)	133 (90.5)	93 (82.3)	34 (13.1)	14 (9.5)	20 (17.7)
CTX	Extended spectrum cephalosporins	226 (86.9)	133 (90.5)	93 (82.3)	1 (0.4)	1 (0.7)	0	33 (12.7)	13 (8.8)	20 (17.7) *
CRO		226 (86.9)	133 (90.5)	93 (82.3)	0	0	0	34 (13.1)	14 (9.5)	20 (17.7)
CAZ		232 (89.2)	137 (93.2)	95 (84.1)	6 (2.3)	1 (0.7)	5 (4.4)	22 (8.5)	9 (6.1)	13 (11.5)
FEP		234 (90.0)	138 (93.9)	96 (85.0)	8 (3.1)	3 (2.0)	5 (4.4)	18 (6.9)	6 (4.1)	12 (10.6) *
ETP	Carbapenems	260 (100)	147 (100)	113 (100)	0	0	0	0	0	0
IPM		260 (100)	147 (100)	113 (100)	0	0	0	0	0	0
MEM		260 (100)	147 (100)	113 (100)	0	0	0	0	0	0
ATM	Monobactams	227 (87.3)	133 (90.5)	94 (83.2)	13 (5.0)	7 (4.8)	6 (5.3)	20 (7.7)	7 (4.8)	13 (11.5) *
AN	Aminoglycosides	260 (100)	147 (100)	113 (100)	0	0	0	0	0	0
GM		255 (98.1)	146 (99.3)	109 (96.5)	0	0	0	5 (1.9)	1 (0.7)	4 (3.5)
NN		257 (98.8)	147 (100)	110 (97.3)	0	0	0	3 (1.2)	0	3 (2.7)
CIP	Fluoroquinolones	238 (91.5)	140 (95.2)	98 (86.7)	3 (1.2)	1 (0.7)	2 (1.8)	19 (7.3)	6 (4.1)	13 (11.5) *
LVX		240 (92.3)	140 (95.2)	100 (88.5)	2 (0.8)	2 (1.4)	0	18 (6.9)	5 (3.4)	13 (11.5) *
SXT	Folate pathway inhibitors	183 (70.4)	109 (74.1)	74 (65.5)	0	0	0	77 (29.6)	38 (25.9)	39 (34.5)

AM—ampicillin, AMC—amoxicillin-clavulanic acid, AN—amikacin, ATM—aztreonam, CAZ—ceftazidime, CIP—ciprofloxacin, CRO—ceftriaxone, CTX—cefotaxime, CXMp—cefuroxime-parenteral, ETP—ertapenem, FEP—cefepime, GM—gentamicin, IPM—imipenem, LVX—levofloxacin, MEM—meropenem, NN—tobramycin, SXT—trimethoprim-sulfamethoxazole, TZP—piperacillin-tazobactam, and * indicates statistically significant resistance (*p* < 0.05).

**Table 3:** Antimicrobial resistance patterns of *E. coli* isolates.

Antimicrobial Resistance Pattern	Resistant Isolates (*n* = 141) Addis Ababa (*n* = 77); Hossana (*n* = 64)				ESBL-Producing Isolates (*n* = 22) Addis Ababa (*n* = 9); Hossana (*n* = 13)		
	No. of Antimicrobial Groups in Resistance Pattern	No. of Isolates with This Resistance Pattern *n* (%)	No. of Isolates with This Resistance Pattern from Addis Ababa *n* (%)	No. of Isolates with This Resistance Pattern from Hossana *n* (%)	No. of ESBL-Producing Isolates with This Resistance Pattern *n* (%)	No. of ESBL Isolates with This Resistance Pattern from Addis Ababa *n* (%)	No. of ESBL Isolates with This Resistance Pattern from Hossana *n* (%)
AM	1	30 (21.3)	16 (20.8)	14 (21.9)	0	0	0
SXT	1	4 (2.8)	2 (2.6)	2 (3.1)	0	0	0
AM, SXT	2	37 (26.2)	22 (28.6)	15 (23.4)	0	0	0
AM, AMC	2	20 (14.2)	17 (22.1)	3 (4.7)	0	0	0
AM, AMC, TZP	3	1 (0.7)	1 (1.3)	0	0	0	0
AM, AMC, SXT	3	7 (5.0)	3 (3.9)	4 (6.3)	0	0	0
AM, CIP, LVX, SXT	3	5 (3.5)	1 (1.3)	4 (6.3)	0	0	0
AM, SXT, CXMp, CRO	4	1 (0.7)	1 (1.3)	0	1 (4.5)	1 (11.1)	0
AM, AMC, GM, SXT	4	1 (0.7)	0	1 (1.6)	0	0	0
AM, ATM, SXT, CAZ	4	1 (0.7)	1 (1.3)	0	0	0	0
AM, GM, CXMp, CTX, CRO	4	1 (0.7)	0	1 (1.6)	1 (4.5)	0	1 (7.7)
AM, SXT, CXMp, CTX, CRO	4	4 (2.8)	2 (2.6)	2 (3.1)	2 (9.1)	1 (11.1)	1 (7.7)
AM, CIP, GM, LVX, NN	3	1 (0.7)	0	1 (1.6)	0	0	0
AM, CXMp, CTX, CRO, FEP	3	1 (0.7)	0	1 (1.6)	1 (4.5)	0	1 (7.7)
AM, SXT, GM, CTX, CXMp, CRO	5	1 (0.7)	1 (1.3)	0	1 (4.5)	1 (11.1)	0
AM, AMC, CXMp, CTX, CRO, FEP	4	2 (1.4)	1 (1.3)	1 (1.6)	2 (9.1)	1 (11.1)	1 (7.7)
AM, ATM, CXMp, CTX, CRO, CAZ	4	2 (1.4)	1 (1.3)	1 (1.6)	0	0	0
AM, SXT, CXMp, CTX, CRO, CAZ	4	2 (1.4)	1 (1.3)	1 (1.6)	1 (4.5)	0	1 (7.7)
AM, CIP, LVX, CXMp, CTX, CRO	4	1 (0.7)	1 (1.3)	0	1 (4.5)	1 (11.1)	0
AM, SXT, CXMp, CTX, CRO, CAZ, FEP	4	1 (0.7)	1 (1.3)	0	0	0	0
AM, ATM, SXT, CXMp, CTX, CRO, CAZ	5	2 (1.4)	0	2 (3.2)	2 (9.1)	0	2 (15.4)
AM, ATM, CXMp, CTX, CRO, CAZ, FEP	4	1 (0.7)	0	1 (1.6)	1 (4.5)	0	1 (7.7)
AM, ATM, SXT, CXMp, CTX, CRO, CAZ, FEP	5	3 (2.1)	1 (1.3)	2 (3.2)	2 (9.1)	1 (11.1)	1 (7.7)
AM, ATM, CIP, LVX, CXMp, CTX, CRO, FEP	5	1 (0.7)	0	1 (1.6)	1 (4.5)	0	1 (7.7)
AM, AMC, SXT, CIP, LVX, CXMp, CTX, CRO	6	1 (0.7)	0	1 (1.6)	0	0	0
AM, ATM, CIP, LVX, CXMp, CTX, CRO, CAZ, FEP	5	1 (0.7)	0	1 (1.6)	0	0	0
AM, AMC, TZP, ATM, CXMp, CTX, CRO, CAZ, FEP	6	1 (0.7)	1 (1.3)	0	1 (4.5)	1 (11.1)	0
AM, AMC, ATM, CIP, LVX, CXMp, CTX, CRO, CAZ, FEP	6	1 (0.7)	0	1 (1.6)	0	0	0
AM, ATM, SXT, CIP, LVX, CXMp, CTX, CRO, CAZ, FEP	6	3 (2.1)	2 (2.6)	1 (1.6)	3 (13.6)	2 (22.2)	1 (7.7)
AM, AMC, ATM, SXT, CIP, LVX, CXMp, CTX, CRO, CAZ	7	1 (0.7)	1 (1.3)	0	0	0	0
AM, AMC, TZP, ATM, SXT, CIP, LVX, CXMp, CTX, CRO, CAZ, FEP	8	1 (0.7)	0	1 (1.6)	1 (4.5)	0	1 (7.7)
AM, AMC, ATM, SXT, CIP, LVX, NN, CXMp, CTX, CRO, CAZ, FEP	8	1 (0.7)	0	1 (1.6)	1 (4.5)	0	1 (7.7)
AM, AMC, ATM, SXT, CIP, LVX, GM, NN, CXMp, CTX, CRO, CAZ, FEP	8	1 (0.7)	0	1 (1.6)	0	0	0

AM—ampicillin, AMC—amoxicillin-clavulanic acid, ATM—aztreonam, CAZ—ceftazidime, CIP—ciprofloxacin, CRO—ceftriaxone, CTX—cefotaxime, CXMp—cefuroxime-parenteral, FEP—cefepime, GM—gentamicin, LVX—levofloxacin, NN—tobramycin, SXT—trimethoprim-sulfamethoxazole, and TZP—piperacillin-tazobactam.

**Table 4:** Phenotypic resistance pattern and associated genetic markers among ESBL-producing *E. coli* isolates.

Strain Designation	Pattern of ESBL Phenotype	Type of β-Lactamase Genes Detected	Origin of Isolate
13	AM, ATM, SXT, CXMp, CTX, CRO, CAZ, FEP	*bla*_TEM-1B_, *bla*_CTX-M-15_	Hossana
39	AM, SXT, CXMp, CRO	*bla*_CTX-M-15_	Addis Ababa
63	AM, SXT, CXMp, CTX, CRO	*bla*_CTX-M-15_	Addis Ababa
69	AM, CIP, LVX, CXMp, CTX, CRO	*bla*_CTX-M-15_	Addis Ababa
75	AM, AMC, TZP, ATM, SXT, CIP, LVX, CXMp, CTX, CRO, CAZ, FEP	*bla*_TEM-1B_, *bla*_CTX-M-15_, *bla*_TEM-169_	Hossana
85	AM, AMC, CXMp, CTX, CRO, FEP	*bla*_CTX-M-3_	Addis Ababa
86	AM, ATM, SXT, CIP, LVX, CXMp CTX, CRO, CAZ, FEP	*bla*_CTX-M-15_	Addis Ababa
92	AM, SXT, CXMp, CTX, CRO, CAZ	*bla*_TEM-1B_, *bla*_CTX-M-15_	Addis Ababa
197	AM, ATM, SXT, CXMp, CTX, CRO, CAZ	#	Hossana
200	AM, AMC, TZP, ATM, CXMp CTX, CRO, CAZ, FEP	#	Addis Ababa
205	AM, ATM, SXT, CIP, LVX, CXMp CTX, CRO, CAZ, FEP	*bla*_TEM-1B_, *bla*_CTX-M-15_	Addis Ababa
232	AM, GM, CXMp, CTX, CRO	*bla*_TEM-1B_, *bla*_CTX-M-15_	Hossana
244	AM, ATM, SXT, CXMp, CTX, CRO, CAZ	*bla*_TEM-1B_	Hossana
260	AM, ATM, SXT, CXMp, CTX, CRO, CAZ, FEP	*bla*_TEM-1B_, *bla*_CTX-M-15_	Hossana
268	AM, ATM, SXT, CIP, LVX, CXMp, CTX, CRO, CAZ, FEP	#	Hossana
289	AM, GM, SXT, CXMp, CTX, CRO	*bla*_TEM-1B_, *bla*_CTX-M-15_	Addis Ababa
302	AM, SXT, CXMp, CTX, CRO	*bla*_TEM-1B_, *bla*_CTX-M-15_	Hossana
303	AM, ATM, CXMp, CTX, CRO, CAZ, FEP	*bla*_TEM-1B_, *bla*_CTX-M-15_	Hossana
306	AM, AMC, ATM, SXT, CIP, LVX, NN, CXMp, CTX, CRO, CAZ, FEP	*bla*_OXA-1_, *bla*_TEM-1B_, *bla*_CTX-M-15_	Hossana
316	AM, ATM, CIP, LVX, CXMp, CTX, CRO, FEP	*bla*_TEM-169_, *bla*_CTX-M-15_	Hossana
324	AM, AMC, CXMp, CTX, CRO, FEP	*bla*_CTX-M-3_	Hossana
340	AM, CXMp, CTX, CRO, FEP	*bla*_CTX-M-3_	Hossana

# Strain was not sequenced as it could not be revived.

## Data Availability

The generated sequencing raw data and assembled genomes were submitted to SRA—Sequence Read Archive (accession number: PRJNA1055930), URL https://www.ncbi.nlm.nih.gov/bioproject/1055930 (accessed on 17 January 2024).

## Supplementary Materials

The following supporting information can be downloaded at: https://www.mdpi.com/article/10.3390/antibiotics13010093/s1, Table S1: Results of antimicrobial susceptibility testing of 260 *Escherichia coli* isolates.

## References

[1] Centers for Disease Control and Prevention (2019). Antibiotic Resistance Threats in The United States 2019.

[2] Urban-Chmiel R., Marek A., ´n-Py´sniak D.S., Wieczorek K., Dec M., Nowaczek A., Osek J. (2022). Antibiotic resistance in bacteria—A review. Antibiotics.

[3] World Health Organization (2022). Global Antimicrobial Resistance and Use Surveillance System (GLASS) Report 2022.

[4] Murray C.J., Ikuta K.S., Sharara F., Swetschinski L., Robles Aguilar G., Gray A., Han C., Bisignano C., Rao P., Wool E. (2022). Global burden of bacterial antimicrobial resistance in 2019: A systematic analysis. Lancet.

[5] Poirel L., Madec J.-Y., Lupo A., Schink A.-K., Skieffer N., Nordmann P., Schwarz S. (2018). Antimicrobial resistance in *Escherichia coli*. Microbiol. Spectr..

[6] World Health Organization WHO Global Priority Pathogens List of Antibiotic-Resistant Bacteria 2021. https://www.doherty.edu.au/news-events/news/who-global-priority-pathogens-list-of-antibiotic-resistant-bacteria.

[7] Tuem K.B., Gebre A.K., Atey T.M., Bitew H., Yimer E.M., Berhe D.F. (2018). Drug resistance patterns of *Escherichia coli* in Ethiopia: A Meta-Analysis. Biomed. Res. Int..

[8] Berhe D.F., Beyene G.T., Seyoum B., Gebre M., Haile K., Tsegaye M., Boltena M.T., Tesema E., Kibret T.C., Biru M. (2021). Prevalence of antimicrobial resistance and its clinical implications in Ethiopia: A systematic review. Antimicrob. Resist. Infect. Control.

[9] Van Hoek A.H.A.M., Mevius D., Guerra B., Mullany P., Roberts A.P., Aarts H.J.M. (2011). Acquired antibiotic resistance genes: An overview. Front. Microbiol..

[10] Bush K., Bradford P.A. (2020). Epidemiology of β-lactamase-producing pathogens. Clin. Microbiol. Rev..

[11] Kiros T., Workineh L., Tiruneh T., Eyayu T., Damtie S., Belete D. (2021). Prevalence of extended-spectrum β-lactamase-producing *Enterobacteriaceae* in Ethiopia: A systematic review and meta-analysis. Int. J. Microbiol..

[12] Poirel L., Naas T., Nordmann P. (2008). Genetic support of extended-spectrum β-lactamases. Clin. Microbiol. Infect..

[13] Ndir A., Diop A., Ka R., Faye P.M., Dia-badiane N.M., Ndoye B., Astagneau P. (2016). Infections caused by extended-spectrum beta-lactamases producing *Enterobacteriaceae*: Clinical and economic impact in patients hospitalized in 2 teaching hospitals in Dakar, Senegal. Antimicrob. Resist. Infect. Control.

[14] Abayneh M., Worku T. (2020). Prevalence of multidrug-resistant and extended-spectrum beta-lactamase (ESBL)-producing gram-negative bacilli: A meta-analysis report in Ethiopia. Drug Target Insights.

[15] Abayneh M., Zeynudin A., Tamrat R., Tadesse M., Tamirat A. (2023). Drug resistance and extended-spectrum β-lactamase (ESBLs)—producing *Enterobacteriaceae*, *Acinetobacter* and *Pseudomonas* species from the views of one-health approach in Ethiopia: A systematic review and meta-analysis. One Health Outlook.

[16] Tufa T.B., Fuchs A., Tufa T.B., Stötter L., Kaasch A.J., Feld T., Häussinger D., Mackenzie C.R. (2020). High rate of extended-spectrum beta-lactamase-producing gram-negative infections and associated mortality in Ethiopia: A systematic review and meta-analysis. Antimicrob. Resist. Infect. Control.

[17] Salleh M.Z., Nik Zuraina N.M.N., Hajissa K., Ilias M.I., Deris Z.Z. (2022). Prevalence of multidrug-resistant diarrheagenic *Escherichia coli* in Asia: A systematic review and meta-analysis. Antibiotics.

[18] Ayukekbong J.A., Ntemgwa M., Atabe A.N. (2017). The threat of antimicrobial resistance in developing countries: Causes and control strategies. Antimicrob. Resist. Infect. Control.

[19] Wolde A., Deneke Y., Sisay T., Mathewos M. (2022). Molecular characterization and antimicrobial resistance of pathogenic *Escherichia coli* strains in children from Wolaita Sodo, Southern Ethiopia. J. Trop. Med..

[20] Adenipekun E.O., Jackson C.R., Ramadan H., Iwalokun B.A., Kolawole S., Frye J.G., Barrett J.B., Hiott L.M., Woodley T.A., Oluwadun A. (2016). Prevalence and multidrug resistance of *Escherichia coli* from community- acquired infections in Lagos, Nigeria. J. Infect. Dev. Ctries.

[21] Mwansa M., Mukuma M., Mulilo E., Kwenda G., Mainda G., Yamba K., Bumbangi F.N., Muligisa-Muonga E., Phiri N., Silwamba I. (2023). Determination of antimicrobial resistance patterns of *Escherichia coli* isolates from farm workers in broiler poultry production and assessment of antibiotic resistance awareness levels among poultry farmers in Lusaka, Zambia. Front. Public Health.

[22] Kebenei K.C., Bett P.K., Onyango P.O., Onyango D.M., Ayieko C., Ang’ienda P.O. (2016). Epidemiology of antimicrobial resistance among *Escherichia coli* strains in Trans-Nzoia County, Kenya. J. Microbiol. Infect. Dis..

[23] Mkuhlu N.A., Chuks I.B., Chikwelu O.L. (2020). Characterization and antibiotic susceptibility profiles of pathogenic *Escherichia coli* isolated from diarrhea samples within the Buffalo City Metropolitan Municipality, Eastern Cape, South Africa. Open Microbiol. J..

[24] Pouwels K.B., Muller-Pebody B., Smieszek T., Hopkins S., Robotham J.V. (2019). Selection and co-selection of antibiotic resistances among *Escherichia coli* by antibiotic use in primary care: An ecological analysis. PLoS ONE.

[25] Pouwels K.B., Freeman R., Muller-Pebody B., Rooney G., Henderson K.L., Robotham J.V., Smieszek T. (2018). Association between use of different antibiotics and trimethoprim resistance: Going beyond the obvious crude association. J. Antimicrob. Chemother..

[26] Negeri A.A., Seyoum E.T., Ibrahim R., Mamo H. (2019). Antimicrobial resistance profile of *Escherichia coli* isolates recovered from diarrheic patients at Selam Health Center, Addis Ababa, Ethiopia. Afr. J. Microbiol. Res..

[27] Ibrahim M.E., Bilal N.E., Hamid M.E. (2012). Increased multi-drug resistant *Escherichia coli* from hospitals in Khartoum state, Sudan. Afr. Health Sci..

[28] Worku F., Tewahido D. (2018). Retrospective assessment of antibiotics prescribing at public primary healthcare facilities in Addis Ababa, Ethiopia. Interdiscip. Perspect. Infect. Dis..

[29] Elmahi O.K.O., Balla S.A., Khalil H.A. (2020). Self-medication with antibiotics and its predictors among the population in Khartoum Locality, Khartoum State, Sudan in 2018. Int. J. Trop. Dis. Health.

[30] Elmahi O.K.O., Musa R.A.E., Shareef A.A.H., Omer M.E.A., Elmahi M.A.M., Altamih R.A.A., Mohamed R.I.H., Alsadig T.F.M. (2022). Perception and practice of self-medication with antibiotics among medical students in Sudanese universities: A cross-sectional study. PLoS ONE.

[31] Chastel C., Mapanguy M., Adedoja A. (2021). High prevalence of antibiotic-resistant *Escherichia coli* in Congolese students. Int. J. Infect. Dis..

[32] Sina H., Dah-Nouvlessounon D., Adjobimey T., Boya B., Dohoue G.M.C., N’tcha C., Chidikofan V., Baba-Moussa F., Abdoulaye I., Adjanohoun A. (2022). Characteristics of *Escherichia coli* isolated from intestinal microbiota children of 0–5 years old in the Commune of Abomey-Calavi. J. Pathog..

[33] Aworh M.K., Kwaga J.K.P., Hendriksen R.S., Okolocha E.C., Harrell E., Thakur S. (2023). Quinolone-resistant *Escherichia coli* at the interface between humans, poultry and their shared environment—A potential public health risk. One Health Outlook.

[34] Adesokan H.K., Akanbi I.O., Akanbi I.M., Obaweda R.A. (2015). Pattern of antimicrobial usage in livestock animals in South-Western Nigeria: The need for alternative plans. Onderstepoort. J. Vet. Res..

[35] Tan X., Kim H.S., Baugh K., Huang Y., Kadiyala N., Wences M., Singh N., Wenzler E., Bulman Z.P. (2021). Therapeutic options for metallo-β-lactamase-producing *Enterobacterales*. Infect. Drug Resist..

[36] Abdelwahab R., Yasir M., Godfrey R.E., Christie G.S., Element S.J., Saville F., Hassan E.A., Ahmed E.H., Abu-Faddan N.H., Daef E.A. (2021). Antimicrobial resistance and gene regulation in Enteroaggregative *Escherichia coli* from Egyptian children with diarrhoea: Similarities and differences. Virulence.

[37] World Health Organization (2019). Critically Important Antimicrobials for Human Medicine: Ranking of Medically Important Antimicrobials for Risk Management of Antimicrobial Resistance Due to Non-Human Use.

[38] Mitman S.L., Amato H.K., Saraiva-Garcia C., Loayza F., Salinas L., Kurowski K., Marusinec R., Paredes D., Cárdenas P., Trueba G. (2022). Risk factors for third-generation spectrum β-lactamase-producing *Escherichia coli* carriage in domestic animals of semirural parishes east of Quito, Ecuador. PLoS Glob. Public Health.

[39] Falgenhauer L., Imirzalioglu C., Oppong K., Akenten C.W., Hogan B., Krumkamp R., Poppert S., Levermann V., Schwengers O., Sarpong N. (2019). Detection and characterization of ESBL-producing *Escherichia coli* from humans and poultry in Ghana. Front. Microbiol..

[40] Beyene A.M., Gezachew M., Mengesha D., Yousef A., Gelaw B. (2022). Prevalence and drug resistance patterns of Gram-negative enteric bacterial pathogens from diarrheic patients in Ethiopia: A systematic review and meta-analysis. PLoS ONE.

[41] Manyi-Loh C., Mamphweli S., Meyer E., Okoh A. (2018). Antibiotic use in agriculture and its consequential resistance in environmental sources: Potential public health implications. Molecules.

[42] Gemeda B.A., Amenu K., Magnusson U., Dohoo I., Hallenberg G.S., Alemayehu G., Desta H., Wieland B. (2020). Antimicrobial use in extensive smallholder livestock farming systems in Ethiopia: Knowledge, attitudes, and practices of livestock keepers. Front. Vet. Sci..

[43] Bevan E.R., Jones A.M., Hawkey P.M. (2017). Global epidemiology of CTX-M β-lactamases: Temporal and geographical shifts in genotype. J. Antimicrob. Chemother..

[44] Blanco J., Demarty R., Park Y., Lavigne J., Alonso M.P., Canic M.M., Park Y.-J., Lavigne J.-P., Pitout J., Johnson J.R. (2008). Intercontinental emergence of *Escherichia coli* clone O25: H4-ST131 producing CTX-M-15. J. Antimicrob. Chemother..

[45] Sewunet T., Asrat D., Woldeamanue Y., Ny S., Westerlund F., Aseffa A., Giske C.G. (2022). Polyclonal spread of blaCTX-M-15 through high-risk clones of *Escherichia coli* at a tertiary hospital in Ethiopia. J. Glob. Antimicrob. Resist..

[46] Tellevik M.G., Blomberg B., Kommedal Ø., Maselle S.Y., Langeland N., Moyo S.J. (2016). High prevalence of faecal carriage of esbl-producing *Enterobacteriaceae* among children in Dar es Salaam, Tanzania. PLoS ONE.

[47] Sangare S.A., Rondinaud E., Maataoui N., Maiga A.I., Guindo I., Maiga A., Camara N., Dicko O.A., Dao S., Diallo S. (2017). Very high prevalence of extended-spectrum beta-lactamase-producing *Enterobacteriaceae* in bacteriemic patients hospitalized in teaching hospitals in Bamako, Mali. PLoS ONE.

[48] Rafaï C., Frank T., Manirakiza A., Gaudeuille A., Mbecko J.R., Nghario L., Serdouma E., Tekpa B., Garin B., Breurec S. (2015). Dissemination of IncF-type plasmids in multiresistant CTX-M-15-producing *Enterobacteriaceae* isolates from surgical-site infections in Bangui, Central African Republic. BMC Microbiol..

[49] Zenebe T., Eguale T., Desalegn Z., Beshah D., Gebre-Selassie S., Mihret A., Abebe T. (2023). Distribution of ß-lactamase genes among multidrug-resistant and extended-spectrum ß-lactamase-producing diarrheagenic *Escherichia coli* from under-five children in Ethiopia. Infect. Drug Resist..

[50] Dela H., Egyir B., Majekodunmi A.O., Behene E., Yeboah C., Ackah D., Bongo R.N.A., Bonfoh B., Zinsstag J., Bimi L. (2022). Diarrhoeagenic, *E. coli* occurrence and antimicrobial resistance of extended spectrum beta-lactamases isolated from diarrhoea patients attending health facilities in Accra, Ghana. PLoS ONE.

[51] Rodríguez I., Thomas K., Van Essen A., Schink A.K., Day M., Chattaway M., Wu G., Mevius D., Helmuth R., Guerra B. (2014). Chromosomal location of *bla*_CTX-M_ genes in clinical isolates of *Escherichia coli* from Germany, the Netherlands and the UK. Int. J. Antimicrob. Agents.

[52] Pitout J.D.D., Gregson D.B., Campbell L., Laupland K.B. (2009). Molecular characteristics of extended-spectrum-β-lactamase-producing *Escherichia coli* isolates causing bacteremia in the calgary health region from 2000 to 2007: Emergence of clone ST131 as a cause of community-acquired infections. Antimicrob. Agents Chemother..

[53] Park S.H., Byun J., Choi S., Lee D., Kim S., Kwon J., Park C., Choi J.-H., Yoo J.-H. (2012). Molecular epidemiology of extended-spectrum β -lactamase-producing *Escherichia coli* in the community and hospital in Korea: Emergence of ST131 producing CTX-M-15. BMC Infect. Dis..

[54] Ouedraogo A.S., Sanou M., Kissou A., Sanou S., Solaré H., Kaboré F., Poda A., Aberkane S., Bouzinbi N., Sano I. (2016). High prevalence of extended-spectrum ß-lactamase producing *Enterobacteriaceae* among clinical isolates in Burkina Faso. BMC Infect. Dis..

[55] Lonchel C.M., Meex C., Gangoué-Piéboji J., Boreux R., Assoumou M.C.O., Melin P., De Mol P. (2012). Proportion of extended-spectrum ß-lactamase-producing *Enterobacteriaceae* in community setting in Ngaoundere, Cameroon. BMC Infect. Dis..

[56] Abrar S., Ain N.U., Liaqat H., Hussain S., Rasheed F., Riaz S. (2019). Distribution of *bla*_CTX-M_, *bla*_TEM_, *bla*_SHV_ and *bla*_OXA_ genes in extended-spectrum-β-lactamase-producing clinical isolates: A three-year multi-center study from Lahore, Pakistan. Antimicrob. Resist. Infect. Control.

[57] Beyene A., Hailu T., Faris K., Kloos H. (2015). Current state and trends of access to sanitation in Ethiopia and the need to revise indicators to monitor progress in the Post-2015 era Global health. BMC Public Health.

[58] Matuschek E., Brown D.F.J., Kahlmeter G. (2014). Development of the EUCAST disk diffusion antimicrobial susceptibility testing method and its implementation in routine microbiology laboratories. Clin. Microbiol. Infect..

[59] The European Committee on Antimicrobial Susceptibility Testing (2023). Breakpoint Tables for Interpretation of MICs and Zone Diameters.

[60] Magiorakos A.P., Srinivasan A., Carey R.B., Carmeli Y., Falagas M.E., Giske C.G., Harbarth S., Hindler J.F., Kahlmeter G., Olsson-Liljequist B. (2012). Multidrug-resistant, extensively drug-resistant and pandrug-resistant bacteria: An international expert proposal for interim standard definitions for acquired resistance. Clin. Microbiol. Infect..

[61] Giske C.G., Martinez- L., Martinez Cantón R., Stefani S., Skov R., Glupczynski Y., Nordmann P., Wootton M., Miriagou V., Simonsen G.S. (2017). EUCAST Guidelines for Detection of Resistance Mechanisms and Specific Resistances of Clinical and/or Epidemiological Importance. http://www.eucast.org/resistance_mechanisms/.

[62] QIAGEN (2016). Blood Mini Handbook QIAGEN Sample and Assay Technologies.

[63] Seegene (2020). STARMag 96 × 4 Universal Cartridge Kit User Manual.

[64] Illumina (2020). Illumina DNA Prep Reference Guide.

[65] Bolger A.M., Lohse M., Usadel B. (2014). Trimmomatic: A flexible trimmer for Illumina sequence data. Bioinformatics.

[66] Babraham Bioinformatics FastQC a Quality Control Tool for High Throughput Sequence Data n.d. https://www.bioinformatics.babraham.ac.uk/projects/fastqc/.

[67] Prjibelski A., Antipov D., Meleshko D., Lapidus A., Korobeynikov A. (2020). Using SPAdes de novo assembler. Curr. Protoc. Bioinform..

[68] Mikheenko A., Prjibelski A., Saveliev V., Antipov D., Gurevich A. (2018). Versatile genome assembly evaluation with QUAST-LG. Bioinformatics.

[69] Florensa A.F., Kaas R.S., Clausen P.T.L.C., Aytan-Aktug D., Aarestrup F.M. (2022). ResFinder—An open online resource for identification of antimicrobial resistance genes in next-generation sequencing data and prediction of phenotypes from genotypes. Microb. Genom..

